# Supplementary material for: Bevacizumab Augments the Antitumor Efficacy of Infigratinib in Hepatocellular Carcinoma
Source: Int J Mol Sci. 2020 Dec 10;21(24):9405. doi: 10.3390/ijms21249405 (PMC7764786; doi:10.3390/ijms21249405)
Supplement: Supplementary file 1 [file ijms-21-09405-s001.pdf]

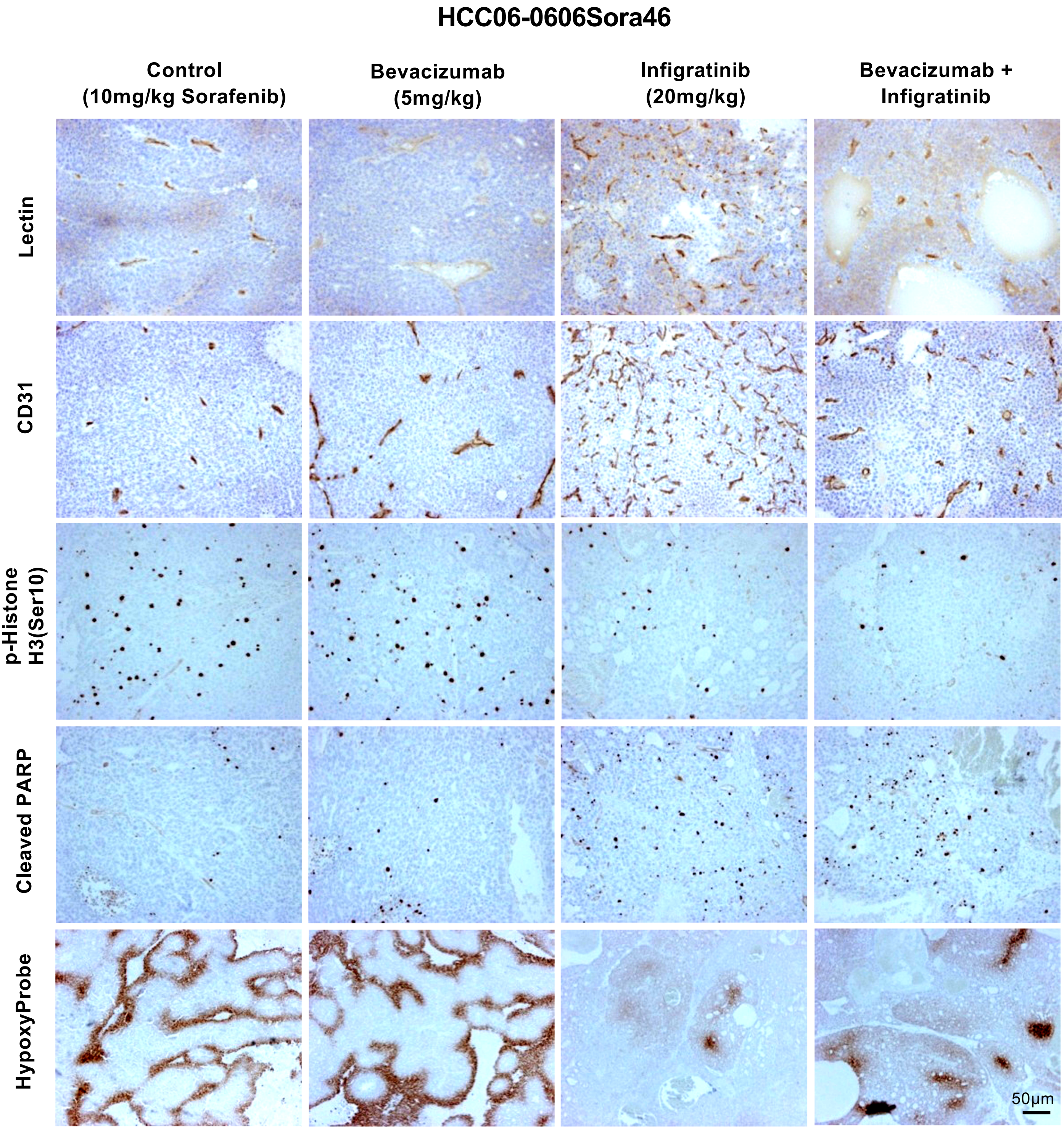

Supplementary Figure 1. Effects of Infigratinib, Bevacizumab and Infigratinib/Bevacizumab on angiogenesis, blood vessel normalization, hypoxia, cell proliferation and apoptosis in HCC06-0606Sora46 model. HCC06-0606Sora46 tumors were subcutaneously implanted into SCID mice. Mice bearing tumor xenografts were treated with 10 mg/kg Sorafenib daily, 20 mg/kg Infigratinib daily, 5 mg/kg Bevacizumab weekly, or 20 mg/kg Infigratinib daily plus 5 mg/kg Bevacizumab weekly for 16 days. Each treatment arm involved 10 independent tumor-bearing mice. Representative pictures of blood vessels stained with anti-CD31, proliferative cells stained with anti-p-Histone H3 Ser10, dead cells stained with anti-cleaved-PARP, functional blood vessels stained with lectin and tumor hypoxia stained with HypoxyProbe antibodies in vehicle- and drug-treated tumors are shown. Bars: 50 μm. Experiments were repeated twice with similar results.

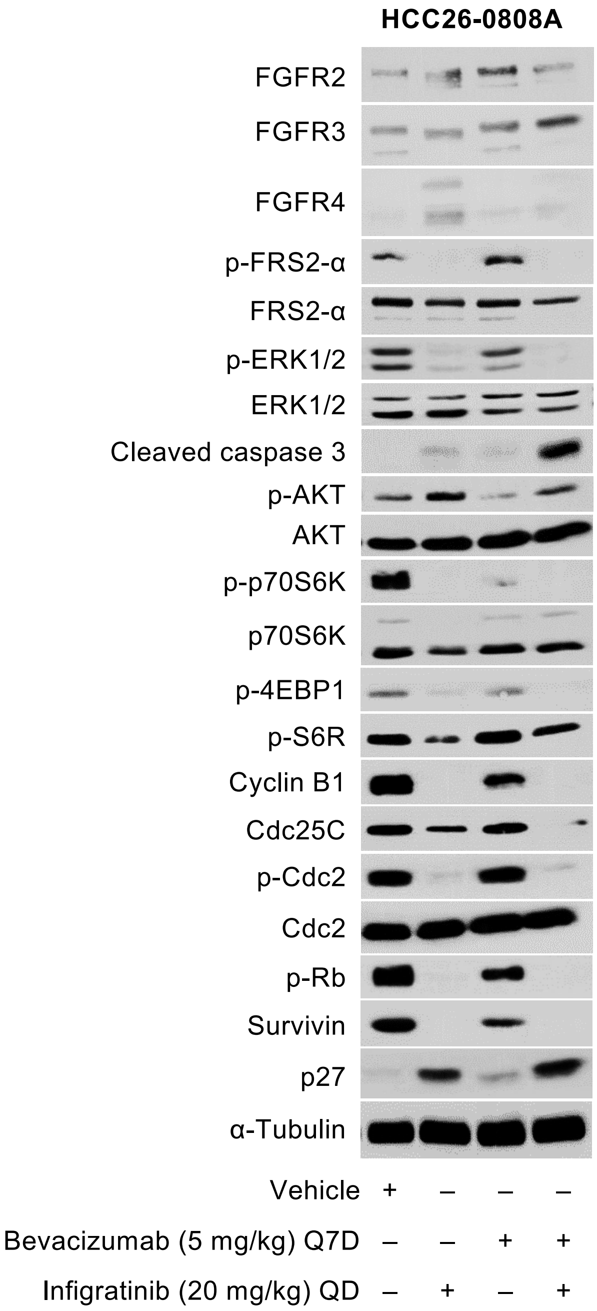

Supplementary Figure 2. Effects of Infigratinib/Bevacizumab on FGFR signaling pathway and its downstream targets in HCC26-0808A model. Mice bearing HCC26-0808A tumors were treated with vehicle, Infigratinib, Bevacizumab, and Infigratinib/Bevacizumab for 5 days as described in Figure 1. Tumors were collected 2 h after the last treatments and tumor lysates subjected to Western blot analysis. Blots were incubated with indicated antibodies and representative blots for HCC26-0808A are shown.

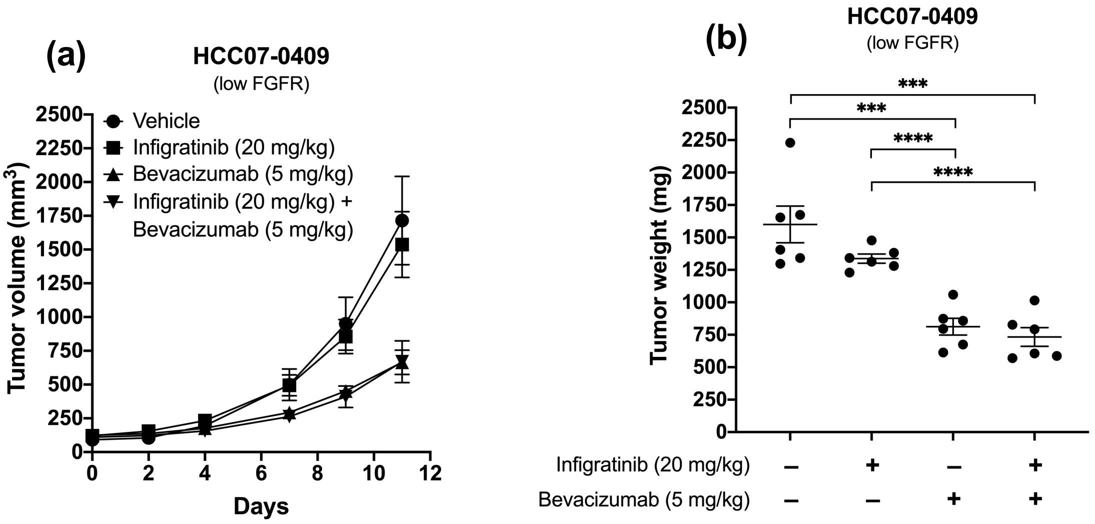

Supplementary Figure 3. Effects of Infigratinib/Bevacizumab on tumor growth in low FGFR-expressing HCC07-0409 models. HCC07-0409 tumors were implanted subcutaneously into SCID mice and subsequently treated as follows: IP injection with 200 μl saline (vehicle), 20 mg/kg Infigratinib, IP injection with 5 mg/kg Bevacizumab, or combined oral Infigratinib and injected Bevacizumab for 12 days. Each group consisted of 6 mice. Tumor volumes were calculated and the mean tumor volumes ± SEs at the indicated time points (a) and tumor weight at sacrifice (b) are shown. Asterisks (*) indicate significant differences ($p < 0.05$, one-way ANOVA with post-hoc Tukey analysis).

HCC13-0109

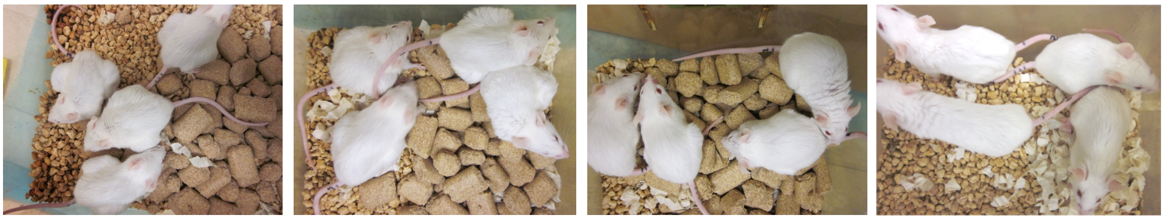

	Vehicle	Bevacizumab (5 mg/kg)	Infigratinib (20 mg/kg)	Infigratinib + Bevacizumab
BUN (mg/dL)	14	15	12	13.5
CRE (mg/dL)	0.47	0.41	0.32	0.32
ALT (U/L)	37.9	39.8	226.5	237.8
ALP (U/L)	57	56.5	82.3	76.3
AST (U/L)	199	208.5	294.8	316.5
TBIL (mg/dL)	0.3	0.3	0.3	0.32
GLU (mg/dL)	152.5	162	159	164
CA (mg/dL)	9.85	9.8	10.45	9.25
TP (g/dL)	5.11	5.15	5.7	5.0
ALB (g/dL)	4.2	4.1	3.85	3.9
GLOB (g/dL)	1.15	1.05	1.05	1.25
Na^+ (mmol/L)	139.5	142.5	136.5	144.5
K^+ (mmol/L)	8.05	8.35	8.7	8.15
Cl^- (mmol/L)	106.3	107.5	105.5	105.5

Supplementary Figure 4. Effects of Infigratinib/Bevacizumab on liver, kidney and hematological injury-related parameters. Mice bearing HCC13-0109 xenografts were treated with vehicle, Infigratinib, Bevacizumab, and Infigratinib/Bevacizumab for 14 days, as described in Figure 1. Serum were collected and analyzed using the Preventive Care Profile Plus (Abaxis, Inc., Union City, CA, USA) according to the manufacturer's instructions. Mice in the treatment groups appear to be in good health. Consistent with the safety profiles of Infigratinib in human studies, daily treatment of mice with Infigratinib resulted in significant elevation in ALT, ALP and AST. Infigratinib also caused a decrease in serum creatinine. The addition of Bevacizumab to Infigratinib did not cause further elevation of serum ALT, ALP, and AST as compared to Infigratinib monotherapy.

Xenograft lines	Angiogenic factor	Vehicle	Bevacizumab (5 mg/kg)	Infigratinib (20 mg/kg)	Infigratinib + Bevacizumab
HCC06-0606	**PDGF-AA**	1	0.94 ± 0.06	0.86 ± 0.12	0.21* ± 0.09
	**VEGF**	1	0.85 ± 0.18	0.45* ± 0.14	0.11** ± 0.07
	**bFGF**	1	0.96 ± 0.14	1.04± 0.16	0.88 ± 0.13
	**HIF-1α**	1	0.96 ± 0.19	0.12* ± 0.04	0.05** ± 0.03
	**CYR61**	1	1.88* ± 0.21	0.98 ± 0.13	0.75** ± 0.11
	**HGF**	1	1.06 ± 0.15	0.98 ± 0.10	1.04 ± 0.11
	**TGF-β1**	1	0.91 ± 0.07	1.02 ± 0.09	0.85 ± 0.10
HCC13-0212	**PDGF-AA**	1	1.04± 0.18	0.98 ± 0.16	1.07± 0.15
	**VEGF**	1	1.82** ± 0.223	0.66* ± 0.15	1.14* ± 0.16
	**bFGF**	1	0.54* ± 0.09	0.56* ± 0.17	0.45* ± 0.08
	**HIF-1α**	1	1.42** ± 0.18	0.35* ± 0.14	0.12*** ± 0.07
	**CYR61**	1	1.66** ± 0.14	0.41* ± 0.18	0.09*** ± 0.05
	**HGF**	1	1.02 ± 0.09	0.98 ± 0.17	0.99 ± 0.10
	**TGF-β1**	1	1.16 ± 0.18	0.96 ± 0.15	0.85 ± 0.22

Supplementary Figure 5. Effects of Infigratinib/Bevacizumab on the expression of angiogenic factors in HCC models. Mice bearing HCC06-0606 and HCC13-0212 xenografts were treated with vehicle, Infigratinib, Bevacizumab, and Infigratinib/Bevacizumab for 14 days, as described in Figure 1. Tumors were collected 2 h after the last treatments and RNA extractions were performed. The levels of VEGF, PDGF-AA, bFGF, CYR61, HIF-1α, VEGF, HGF and TGF-β1 mRNA were determined using the ViiA7 Real-time PCR system. To standardize the RNA level, the expression of GAPDH mRNA in each sample was quantified. The levels of expressed bFGF, PDGF-AA, VEGF, CYR61, TGF-β1, HGF and HIF-1α mRNA were then normalized by that of GAPDH.
